# A Packed-Type Reconfigurable Air Filtration System for Removal of Particulate Matter and HCHO

**DOI:** 10.3390/polym17243312

**Published:** 2025-12-15

**Authors:** Eun Jin Kim, Seung Hee Han, Dong Geon Lee, Won San Choi

**Affiliations:** Department of Chemical and Biological Engineering, Hanbat National University, 125 Dongseo-daero, Yuseong-gu, Daejeon 305-719, Republic of Korea; kimej106@naver.com (E.J.K.); hansh184@naver.com (S.H.H.); ac3958@naver.com (D.G.L.)

**Keywords:** particulate matter, HCHO, air filters, removal efficiency, pressure drop

## Abstract

A flexible and packed-type air filter ball (AFB) system was developed for the efficient removal of particulate matter (PM) and formaldehyde (HCHO). The Mg/Si-embedded AFB (Mg/Si-AFB) was synthesized through sequential physical etching of a sponge, oxidation of glass fiber, and subsequent formation of Mg-Si components. The resulting Mg/Si-AFB exhibited a highly porous and roughened architecture with enhanced surface reactivity. A disk-type filtration device loaded with Mg/Si-AFBs demonstrated a PM_2.5_ removal efficiency (RE) of 97.4% at a pressure drop of 57 Pa. The RE increased with packing density and PM concentration and notably remained constant even at high air velocities (7 m/s). In addition, the oxidized glass fiber (GF)-based AFB (O-GF-AFB) exhibited rapid HCHO adsorption capability, achieving 100% HCHO removal within 1 min. Hybrid air filters combining Mg/Si-AFBs and O-GF-AFBs in an equal ratio (8:8) exhibited synergistic performance, simultaneously achieving 97.1% PM_2.5_ RE and complete HCHO removal within 1–6 min, while maintaining low pressure drops (55–57 Pa) over 50 reuse cycles.

## 1. Introduction

Air pollution remains one of the most pressing global environmental challenges, directly threatening human health and the stability of urban ecosystems. The rapid increase in industrialization, vehicular emissions, and indoor chemical releases has resulted in complex mixtures of airborne contaminants, including particulate matter (PM), volatile organic compounds (VOCs), and toxic gases such as formaldehyde (HCHO), sulfur dioxide, and nitrogen oxides. Depending on their aerodynamic diameters, PM can be categorized as PM_10_, PM_2.5_, and PM_0.3_, which represent particles smaller than 10, 2.5, and 0.3 µm, respectively. Among them, fine and ultrafine PMs such as PM_2.5_ and PM_0.3_ are particularly hazardous because they can penetrate deep into the respiratory system, accumulate in the lungs, and even enter the bloodstream, resulting in cardiovascular and pulmonary diseases [1,2,3,4]. HCHO poses a significant threat to human health, particularly for those primarily spending time indoors, as this highly toxic gas is spontaneously emitted from numerous industrial products. Frequent exposure to HCHO is associated with elevated cancer rates among individuals [5,6]. Prolonged exposure to these pollutants has been associated with respiratory illnesses, cardiovascular diseases, and even carcinogenic effects [7,8,9]. Accordingly, the development of efficient air filtration systems has become an essential technological priority for both outdoor and indoor environments. A variety of hydrophilic air filters have been developed for effective PM removal [10,11,12,13,14,15,16,17,18,19]. Given that PM is primarily composed of highly polar materials [20], the development of these hydrophilic filters is widespread [10,11,12,13,14,15,16,17,18,19]. Furthermore, hybrid air filters combining hydrophilic and hydrophobic components have also been proposed to prevent the formation of water films while simultaneously boosting removal efficiency [21,22]. Recently, water-based air filters have gained attention as a potential solution due to the hydrophilic nature of PM and HCHO [23]. Because these pollutants can be dispersed or dissolved in water, integrating aqueous systems into air filtration could provide a natural and sustainable approach. Recent advances in air purification have highlighted the growing need for modular and reconfigurable filtration systems capable of adapting to diverse indoor and industrial environments. Reviews on hybrid filtration technologies summarize progress in combining mechanical and sorptive/catalytic approaches, but also note trade-offs between removal performance and pressure drop or operational lifetime [24,25]. Recent modular and bead/packed-type filter concepts illustrate practical strategies for customizable filter assemblies and low-pressure-drop devices, but many of these systems remain constrained by fixed frame geometries or limited real-time tunability of packing density and functional combinations [15,26].

Most current systems are designed as static and uniform filter modules that operate under fixed structural and material configurations. While such designs simplify fabrication and operation, they inherently limit the adaptability of the system toward specific pollutants and varying environmental conditions [27]. For instance, filters optimized for PM removal may show limited performance toward gaseous molecules, while those designed for chemical adsorption often exhibit high pressure drops or short operational lifetimes due to pore blockage. Consequently, there is an urgent demand for adaptive and reconfigurable air filtration technologies that can dynamically adjust to the pollutant composition and concentration in the surrounding air. One promising direction involves the concept of customizable air filters, which enable users or operators to selectively target specific pollutants through the integration of tailor-made filtration units. Instead of relying on a single, fixed medium, a customizable system would allow modular insertion of filters with different functionalities—such as PM-capturing fibers, gas-adsorbing catalysts, or antibacterial components—depending on the application environment. This strategy not only enhances filtration selectivity but also maximizes material efficiency by allowing pollutant-specific deployment. To realize such flexibility, the design of the air filtration system plays a pivotal role. The air filtration system must be engineered to allow easy insertion, replacement, and removal of filter elements without disrupting the operation or requiring complex disassembly. If users can freely load and unload a desired quantity of filter material, it becomes possible to precisely control both the filtration efficiency and the pressure drop. Therefore, an air filtration system that permits real-time tuning of filter loading would provide adaptive control over both performance and operational cost.

We report a universal, reconfigurable air filtration platform designed to provide a transformative solution for next-generation purification systems. Such a platform would enable the customized targeting of specific pollutants while providing user-controlled adjustment of filtration efficiency and pressure drop characteristics. The combination of modular design, pollutant-specific functionality, and ease of reconfiguration could open new possibilities for smart, efficient, and sustainable air purification in both residential and industrial applications.

## 2. Experimental Section

### 2.1. Materials

A sulfate melamine–formaldehyde sponge (MFS) was purchased from BASF (Berlin, Germany). Glass fiber (GF) was purchased from Youngnam-Sangsa (Daejeon, Republic of Korea). Sodium hydroxide (NaOH), magnesium chloride hexahydrate (MgCl_2_ · 6H_2_O), ammonium chloride (NH_4_Cl), and ammonium hydroxide solution (NH_4_OH, 28–30%) were purchased from Sigma-Aldrich (Burlington, MA, USA). Sandpaper was purchased from Easyway (Daejeon, Republic of Korea). Deionized (DI) water (resistivity: 18 MΩ cm) was produced using a Millipore (Burlington, MA, USA) Simplicity 185 system.

### 2.2. Preparation of the MFS/GF

MFS/GF composites were prepared by immersing 16 cylindrical MFS samples (diameter: 2 cm, height: 2 cm) into a beaker lined with sandpaper and containing a GF solution (1 g GF dissolved in 300 mL of DI water). The suspension was stirred for 6 h to promote interaction between the spherical MFS and GF. When stirred for an extended period, the loosely entangled and micron-sized GFs become well-suspended in the aqueous medium. This dispersion allows the fibers to interact effectively with the MFS during the mechanical etching process. The obtained composites were then washed three times with DI water and dried in an oven at 50 °C for 12 h to yield the MFS/GF samples.

### 2.3. Preparation of the O-GF-AFB (MFS/O-GF)

A 20 wt% NaOH solution was prepared, and the MFS/GF samples were immersed in the solution for 24 h to obtain MFS/O-GF. The resulting samples were then washed three times with DI water and dried in an oven at 50 °C for 12 h to yield the O-GF-AFB (MFS/O-GF).

### 2.4. Preparation of the Mg/Si-AFB (MFS/O-GF/Mg-Si)

50 mg of MgCl_2_·6H_2_O, 530 mg of NH_4_Cl, and 1 mL of NH_4_OH solution were added to 50 mL of DI water and stirred to obtain a homogeneous mixture. The mixed solution and the MFS/O-GF composite were then placed in a Teflon-lined autoclave and heated at 140 °C for 4 h. The resulting MFS/O-GF/Mg–Si composite was washed three times with DI water and dried in an oven at 70 °C for 12 h. GFs are composed primarily of silicon-oxide–based components (SiO_2_). When the O-GF–containing MFS is subsequently treated in the hydrothermal Mg precursor solution (MgCl_2_·6H_2_O + NH_4_Cl + NH_4_OH), the following sequence occurs: During the NaOH oxidation step, SiO_2_-rich GFs develop abundant surface -OH groups. Under hydrothermal conditions (140 °C, 4 h), Mg^2+^ ions react with the surface-exposed Si-OH groups, promoting in situ formation of Mg-Si species on the O-GF surfaces [28]. This reaction results in the nucleation and growth of rough, porous Mg-Si structures anchored to the embedded O-GF.

### 2.5. PM and HCHO Filtration

PM was generated by burning incense. The PM source chamber (500 mL) was filled with low (PM_2.5_: 490 μg/m^3^), medium (PM_2.5_: 1510 μg/m^3^), or high (PM_2.5_: 641,710 μg/m^3^) concentrations of PM and connected to the input chamber. A constant airflow velocity of 1, 3, 5, or 7 m/s was introduced into the PM source chamber to transfer PM to the filter chamber equipped with the filter. Unfiltered PM passing through the filter was detected by a particle detector located in the output chamber. At very high PM concentrations, particle agglomeration tends to occur; therefore, temporal changes in PM concentration within the reservoir were carefully monitored prior to conducting PM removal tests. To ensure measurement accuracy, each experiment was repeated three times, and the average values were reported. The RE was calculated by comparing the measured PM particle concentrations before and after filtration.

RE (%) = (C_0_ − C_1_)/C_0_ × 100%

where C_0_ (µg m^−3^) and C_1_ (µg m^−3^) are the PM concentrations before and after filtration, respectively. The pressure drop across the filter was measured using a differential pressure meter installed at the inlet and outlet sections of the filter. For HCHO gas removal tests, a 500 mL glass chamber containing 10 μL of liquid HCHO was connected to the input chamber. An airflow (1 m/s) was introduced into the glass chamber to transport the HCHO gas into the input and filter chambers. Unfiltered VOCs emitted from the filter were detected by a gas detector located in the output chamber. The RE was calculated by comparing the HCHO concentrations measured before filtration (detector 1) and after filtration (detector 2). The laser-based PM counter (HT-9601) was calibrated using a zero filter prior to experiment to ensure baseline stability. The formaldehyde detector (BQ16) was calibrated using a certified 1 ppm HCHO standard gas before each measurement cycle. Both instruments were calibrated prior to each experiment following the manufacturer’s recommended protocols.

### 2.6. Characterization

SEM and EDX analyses were conducted on a Hitachi (Tokyo, Japan) S-4800 microscope. Thermogravimetric analysis (TGA) was conducted with a SETARAM Labsys Evo (Caluire, France) TG-DTA analyzer. Fourier transform infrared (FT-IR) spectra were obtained using a Sinco Nicolet IS5 instrument. X-ray photoelectron spectroscopy (XPS) was performed using an Axis NOVA (Kratos Analytical, Manchester, UK) spectrometer using an aluminum anode (Al K-alpha, 1486.6 eV) operated at 600 W. Measurements of HCHO concentrations were performed using HCHO meters (BQ16, Trotec GmbH & Co. KG, Heinsberg, Germany). PM concentrations before and after filtration were measured using a portable air quality detector (HT-9601, Dongguan Xintai Instrument, Dongguan, China). The pressure drop was determined using a differential pressure gauge (TESTO 510i, TESTO, Titisee-Neustadt, Germany), and the flow rate was measured with a flowmeter (TESTO 450i, TESTO, Titisee-Neustadt, Germany).

## 3. Results and Discussion

**Figure 1:**
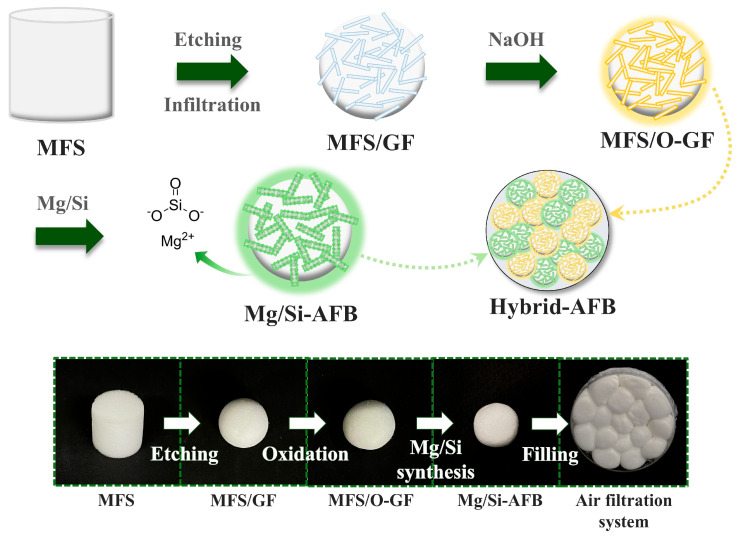
Schematic illustration of the synthesis of the O-GF-AFB (MFS/O-GF) and Mg/Si-AFB (MFS/O-GF/Mg-Si), and a disk-type air filtration system in which the O-GF-AFBs and Mg/Si-AFBs are loaded to capture PM or HCHO, together with their corresponding real images.

Figure 1 schematically illustrates the formation of a Mg/Si-embedded air filter ball (Mg/Si-AFB). The synthesis involves the physical etching of a melamine formaldehyde sponge (MFS), followed by the oxidation of glass fiber (GF) and the subsequent synthesis of the Mg/Si component. Given its porous, compressible, and sulfonated functional properties, MFS was employed as the base material. The Mg/Si-AFB (MFS/O-GF/Mg-Si) is prepared by immersing a cylindrical MFS into a beaker wrapped in sandpaper that contains the GF solution. During stirring, the centimeter-scale cylindrical MFS is gradually transformed into a tiny spherical GF-embedded MFS through collisions involving the MFS, GF, and sandpaper. To achieve the effective removal of PMs and HCHO, a disk-type air filter filled with 12 to 18 Mg/Si-AFBs or O-GF-AFBs was designed. Hybrid AFBs, composed of Mg/Si-AFBs and O-GF-AFBs, are prepared to realize customizable air filters that enable users or operators to selectively target specific pollutants.

**Figure 2:**
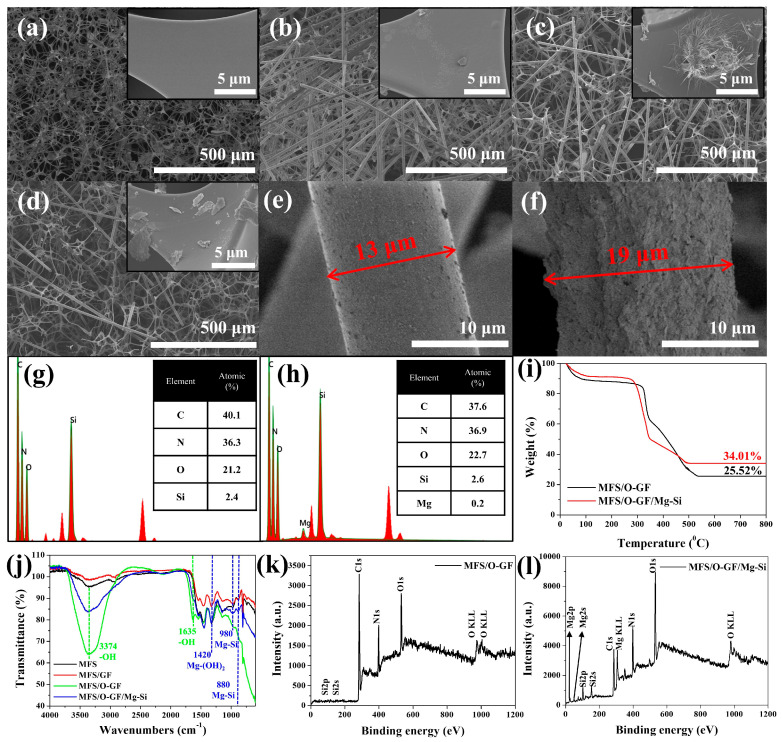
(**a**–**f**) (SEM) images illustrating the morphological changes at each synthesis step: (**a**) MFS, (**b**) MFS/GF, (**c**) MFS/O-GF, (**d**) MFS/O-GF/Mg-Si, (**e**) O-GF component within MFS/O-GF, and (**f**) Mg-Si component within MFS/O-GF/Mg-Si. (**g**,**h**) EDX data for the (**g**) MFS/O-GF composite and (**h**) MFS/O-GF/Mg-Si composite. (**i**) TGA data comparing the MFS/O-GF and MFS/O-GF/Mg-Si composites. (**j**) FT-IR spectroscopy data for the MFS, MFS/GF, MFS/O-GF, and MFS/O-GF/Mg-Si. (**k**,**l**) XPS data for the (**k**) MFS/O-GF composite and (**l**) MFS/O-GF/Mg-Si composite.

Figure 2a–d shows scanning electron microscopy (SEM) images of each step of the Mg/Si-AFB (MFS/O-GF/Mg-Si) formation. The MFS substrate exhibited an interconnected 3D network skeleton with its inherent surface morphology (Figure 2a). After the physical etching of MFS using sandpaper and GF, the GF interpenetrated the MFS pores, and the MFS surfaces became marginally rougher (MFS/GF) (Figure 2b). This change suggests that physical etching of the MFS surface occurred due to friction from the sandpaper and GF during the process. After the oxidation of the MFS/GF composite, the MFS surfaces became significantly rougher, and small particles were observed on the surface of the GF (MFS/O-GF) (Figure 2c,e). Following the synthesis of Mg-Si onto the MFS/O-GF, the width of the O-GF/Mg-Si composite significantly increased from 13 μm to 19 μm (Figure 2e,f). Concurrently, the surface of the O-GF/Mg-Si became remarkably rough and porous (MFS/O-GF/Mg-Si) (Figure 2f). Brunauer–Emmett–Teller (BET) analysis revealed that the MFS/O-GF sample exhibited a specific surface area of 0.3145 m^2^/g, while the MFS/O-GF/Mg–Si (Mg/Si-AFB) showed a higher surface area of 0.4894 m^2^/g (Figure S1). The ~55% increase in surface area is attributed to the Mg–Si nanostructures grown during the hydrothermal treatment, providing additional adsorption sites that contribute to improved filtration and HCHO RE. Energy-dispersive X-ray (EDX) and thermogravimetric analysis (TGA) analyses further confirmed the successful formation of the Mg/Si-AFB. The presence of Si in the MFS/O-GF composite suggested that the O-GF, which contains Si, successfully infiltrated the MFS structure (Figure 2g). While Mg was not detected in the MFS/O-GF composite, its subsequent observation in the MFS/O-GF/Mg-Si composite indicated that the Si present in the MFS/O-GF was successfully transformed into the Mg-Si component within the final composite (Figure 2h). The O-GF content, accounting for 25.52% of the total MFS/O-GF composite mass, supports the successful loading of O-GF onto the MFS (Figure 2i). Furthermore, the mass percentage of the inorganic portion (Mg-Si or Si) increased to 34.01% in the MFS/O-GF/Mg-Si composite, which suggests the transformation of Si into Mg-Si during the synthesis (Figure 2i). Fourier Transform Infrared (FT-IR) spectroscopy was performed to confirm the formation of the Mg/Si-AFB. Following oxidation, the MFS/O-GF composite exhibited new absorption peaks at 3374 cm^−1^ (-OH) and 1635 cm^−1^ (-OH) (Figure 2j, green line), indicating the successful oxidation of GF to O-GF.

Finally, the MFS/O-GF/Mg-Si composite showed new characteristic absorption peaks at 1420 cm^−1^ (Mg-(OH)_2_), 980 cm^−1^ (Mg-Si), and 880 cm^−1^ (Mg-Si) (Figure 2j, blue line) [28]. These peaks confirm the transformation of Si into the Mg-Si component during the synthesis process. X-ray photoelectron spectroscopy (XPS) characterization provided additional evidence for the successful synthesis of the Mg/Si-AFB. The detection of Si 2p signals in the MFS/O-GF composite verified the effective incorporation of Si-containing O-GF into the MFS framework (Figure 2k). In contrast, Mg-related peaks were absent in the MFS/O-GF composite but newly appeared in the MFS/O-GF/Mg-Si composite, confirming the formation of Mg–Si species (Figure 2l). These results indicate that the Si sites originating from O-GF participated in the in situ transformation into Mg–Si components during the subsequent reaction. XRD analysis of the Mg/Si-AFB did not show distinct crystalline Mg–Si peaks; this is attributed to the low surface loading of Mg–Si relative to the dominant amorphous polymer matrix and to the likely poor crystallinity of the hydrothermally formed Mg–Si species (Figure S2). FT-IR and XPS, however, detect Si–O–Mg-related vibrations and Mg-related chemical states, and BET measurements show a ∼55% increase in surface area after Mg–Si treatment (0.3145 → 0.4894 m^2^/g), consistent with formation of nanoscale Mg–Si surface structures. Therefore, the combined dataset supports the formation of surface-localized, poorly crystalline (or amorphous) Mg–Si phases.

**Figure 3:**
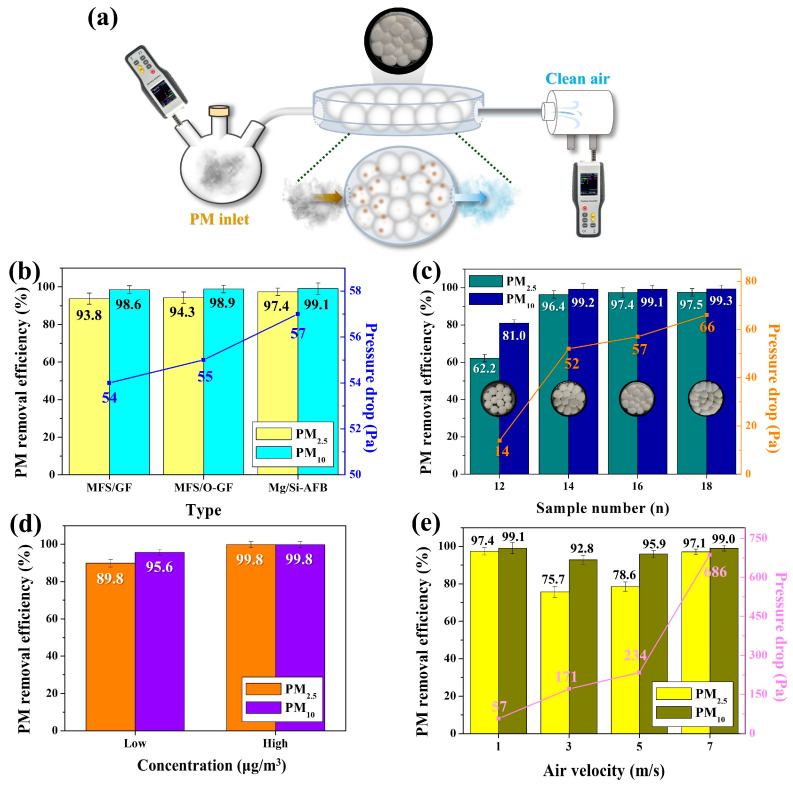
(**a**) Experimental setup of the disk-type air filtration system containing O-GF-AFBs or Mg/Si-AFBs for PM removal. (**b**) PM REs and pressure drops of the MFS/GF, MFS/O-GF, and MFS/O-GF/Mg-Si composites. (**c**) PM REs and pressure drops of the MFS/O-GF/Mg-Si (Mg/Si-AFB) composite as a function of the number of AFBs. (**d**) PM REs of the Mg/Si-AFBs at low and high PM concentrations. (**e**) PM REs and pressure drops of the Mg/Si-AFBs as a function of increasing air velocity.

Before testing the performance of the Mg/Si-AFB, we investigated the PM removal capabilities of the MFS/GF and MFS/O-GF composites. As illustrated in Figure 3a, a disk-type air filtration system loaded with the air filter was connected between the PM source/detector 1 (left) and detector 2 (right). The left chamber contained 500 mL of PM-laden air with 1510 μg/m^3^ of PM_2.5_. A constant air velocity of 1 m/s was applied to the source chamber to force the PM-laden air to flow from the left chamber to the right chamber through each air filter. Unfiltered PM released from the filter was then detected by the detector. To investigate the effects of the filter materials on PM removal efficiency (RE), three types of filters such as MFS/GF, O-GF-AFB (MFS/O-GF), and Mg/Si-AFB (MFS/O-GF/Mg-Si) were tested. The PM_2.5_ RE of MFS/GF was 93.8%, which increased slightly to 94.3% for the O-GF-AFB (MFS/O-GF). This result suggests that the oxidation of GF contributes to improving the PM RE. The PM_2.5_ RE of the Mg/Si-AFB was 97.4%, which was significantly higher than those of the MFS/GF (93.8%) and O-GF-AFB (MFS/O-GF) (94.3%), respectively (Figure 3b). These results suggest that PM preferentially interacts with the Mg/Si surface in the Mg/Si-AFB rather than with the O-GF surface in the O-GF-AFB, likely due to the higher specific surface area of the Mg/Si material, as shown in Figure 2f. The PM_10_ REs of the three filters exhibited minimal variation (98.6~99.1%), which may be attributed to mechanical collection mechanisms such as inertial impaction and interception (ice blue bars, Figure 3b). In contrast, the observed differences in PM_2.5_ RE (93.8~97.4%) could be influenced by factors including diffusion (Brownian motion), electrostatic effects (electret), and the surface area and structural characteristics of the filter materials (yellow bars, Figure 3b). Therefore, the relatively superior performance of Mg/Si-AFB is likely associated with its distinct surface morphology and material properties compared with the other filters. A disk-type air filtration system loaded with 16 Mg/Si-AFBs exhibited the highest pressure drop of 57 Pa, compared with 54 Pa and 55 Pa for the systems containing 16 MFS/GF balls and 16 O-GF-AFBs, respectively (Figure 3b). These results indicate that the Mg/Si-AFB has a denser and more complex structure than the others. Thus, the Mg/Si-AFB, exhibiting a PM_2.5_ RE of 97.4% and a pressure drop of 57 Pa, was selected as the optimized AFB. Figure 3c shows the PM_2.5_ RE and pressure drop of the disk-type air filtration system loaded with different numbers of Mg/Si-AFBs (12, 14, 16, and 18). These systems are denoted as AFB-12, AFB-14, AFB-16, and AFB-18, respectively. The internal void spaces within the disk-type air filtration system decreased and eventually disappeared as the packing density of AFBs increased (inset of Figure 3c). Both the PM_2.5_ RE and pressure drop increased with the packing density of AFBs. While the PM_2.5_ REs of AFB-14 and AFB-16 increased noticeably, that of AFB-18 remained nearly identical to AFB-16, indicating that excessive packing of AFBs does not further enhance PM RE (green bars, Figure 3c). In contrast, excessive packing led to a pronounced increase in the pressure drop of the filtration system. Therefore, the system loaded with 16 Mg/Si-AFBs was selected as the optimized configuration. The packing density of the Mg/Si-AFB was calculated as the total mass of the Mg/Si-AFBs divided by the disk-type filtration device volume (127.4 cm^3^). For the Mg/Si-AFB (MFS/O-GF/Mg-Si), the packing densities were 14.16, 16.52, 18.89, and 21.24 kg/m^3^ when 12, 14, 16, and 18 Mg/Si-AFBs were loaded, respectively, confirming that the filtration module enables precise control of packing density by simply varying the number of AFBs (Figure S3). Because the system adopts a filling-type configuration, the packing density in the air filter chamber can be easily tuned, allowing straightforward adjustment of both removal efficiency and pressure drop through the number of AFBs loaded.

To evaluate the effect of PM concentration on RE, the PM REs of the system loaded with 16 Mg/Si-AFBs were measured at lower and higher PM concentrations (490 and 641,710 µg/m^3^) relative to the standard concentration (1510 µg/m^3^). The 16 Mg/Si-AFB system exhibited PM_2.5_ REs of 89.8% and 99.8% at the lower and higher concentrations, respectively (orange bars, Figure 3d). These observed PM RE values were significantly lower and higher, respectively, than the PM RE measured at the standard concentration. Exposed to high concentrations of PM, the air filters rapidly capture PM particles on their surface and between the fibers, leading to the formation of a dust cake. This accumulated layer of PM particles acts, in itself, as a denser and finer filtration medium. The dust cake more effectively captures smaller particles than the clean filter fibers alone, temporarily increasing the overall RE. Consequently, the filter is rapidly “aged” or conditioned during high-concentration tests, resulting in a higher RE. To investigate the removal performance of our air filtration system under different air velocities, the PM_2.5_ REs were measured as the air velocity increased. Typically, the PM RE decreases with increasing air velocity due to the reduced contact time between airborne particles and the filter, while the pressure drop increases with increasing air velocity. In our system, the pressure drop rose from 57 Pa to 686 Pa as the air velocity increased (pink line, Figure 3e). However, a distinctive pattern in PM RE was observed: the PM_2.5_ RE decreased at an air velocity of 3 m/s, slightly increased at 5 m/s, and then significantly increased at 7 m/s (yellow bars, Figure 3e). The PM_2.5_ and PM_10_ REs (97.1% and 99%, respectively) measured at an air velocity of 7 m/s were nearly identical to those obtained at 1 m/s (Figure 3e). These results suggest that the disk-type air filtration system loaded with 16 Mg/Si-AFBs can maintain a consistent PM RE even under high air velocity conditions. Our reconfigurable air filtration platform allows easy insertion and removal of AFBs as many times as desired within the open/close-type disk chamber. This enables control of the RE and pressure drop of the air filtration system according to environmental conditions, as shown in Figure 3c.

**Figure 4:**
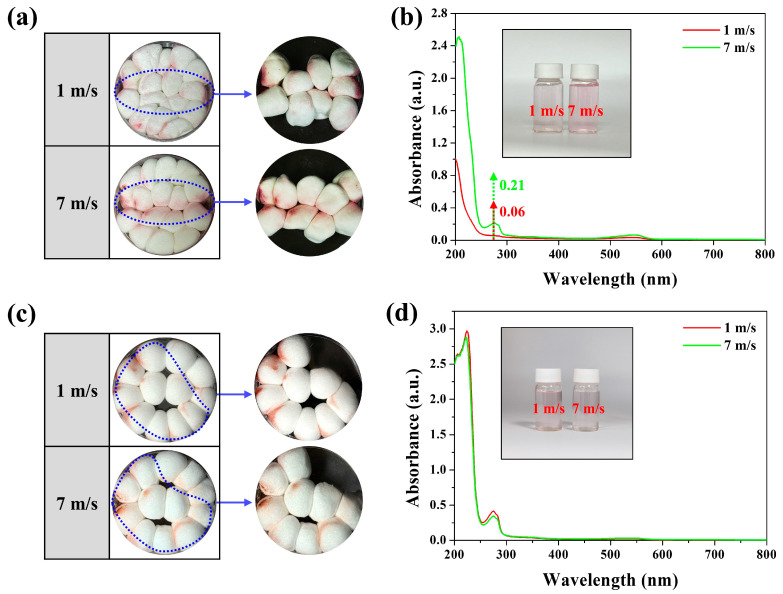
(**a**) Images of the disk-type air filtration system containing 16 Mg/Si-AFBs (high packing density) at different air velocities. (**b**) UV–vis absorption spectra of the PM solutions obtained at 1 and 7 m/s. The inset shows the corresponding solutions. (**c**) Images of the disk-type air filtration system containing 12 Mg/Si-AFBs (low packing density) at different air velocities. (**d**) UV–vis absorption spectra of the PM solutions obtained at 1 and 7 m/s. The inset shows the corresponding solutions.

To clarify the reason why the Mg/Si-AFB maintained a high RE even at high air velocities, a visualization experiment was conducted to trace the movement path of PM-laden air within the filter. Red smoke bombs were used to visualize the PM trajectories, and 16 Mg/Si-AFB samples were used for this test. At an air velocity of 1 m/s, PM was uniformly adsorbed throughout the entire filter, whereas at 7 m/s, PM deposition was concentrated along the inlet and outlet flow paths (Figure 4a). To quantitatively verify whether more PM was adsorbed by the Mg/Si-AFBs located along these paths, nine Mg/Si-AFBs were collected and immersed in water to measure the amount of adsorbed PM. The PM solution obtained from the 7 m/s sample exhibited a darker pink coloration and higher UV–vis absorbance compared to that from the 1 m/s sample, indicating greater PM accumulation along the flow path under high-flow conditions (Figure 4b). To elucidate the effect of packing density on RE, adsorption behavior was examined in an air filtration system with a low packing density (12 Mg/Si-AFB samples). At this lower density, similar adsorption patterns were observed under both low (1 m/s) and high (7 m/s) air velocities, with PM deposition occurring uniformly throughout the filter (Figure 4c). The PM solution obtained at 1 m/s exhibited a more intense pink coloration and slightly higher UV–vis absorbance than that obtained at 7 m/s (Figure 4d). In other words, a slightly higher adsorption amount was detected at the lower air velocity (1 m/s), consistent with the longer residence time of PM-laden air. As expected, the filtration efficiency decreased at high air velocities under low packing density conditions, a typical trend in conventional air filters. However, as shown in Figure 3e and Figure 4a,b, when the packing density was increased (16 Mg/Si-AFB samples), no deterioration in RE was observed even at high air velocities. These findings indicate that maintaining a sufficiently high packing density allows the air filter to sustain high RE even under rapid airflow conditions. To quantitatively evaluate the contribution of the filter cake effect at high airflow velocities, we analyzed the PM mass and performance change between low-packing and high-packing systems. The absorbance of PM extracts at 7 m/s was 0.209 at 273 nm, corresponding to a ~348% higher PM mass compared to 1 m/s (0.06). This indicates rapid formation of a dense filter cake along the inlet–outlet flow path under high airflow (Figure 4b). We also compared the performance change between low-packing (12 Mg/Si-AFBs) and high-packing (16 Mg/Si-AFBs) systems. The PM_2.5_ RE decreased by 8.6% in the low-packing system when airflow increased from 1 to 7 m/s, whereas the high-packing system showed only a 0.3% decrease (Figure S4). This demonstrates that the filter-cake layer plays a dominant role in sustaining filtration efficiency at high flow. To sum up, at a low air velocity, when PM-laden air encounters the filter, part of the flow penetrates while another part bypasses the fibers, allowing sufficient interaction between PM and the entire filter structure (Figure 4a). In contrast, at a high air velocity, most PM-laden air passes directly toward the outlet without sufficient interaction with the filter matrix, resulting in preferential adsorption near the inlet and along the main flow passages (Figure 4a). Consequently, a filter cake forms on the passage side, which further enhances the PM RE (Figure 4b). Therefore, the unique internal architecture arising from the dense packing of AFBs enables the air filter to maintain a high RE even under elevated air velocities.

**Figure 5:**
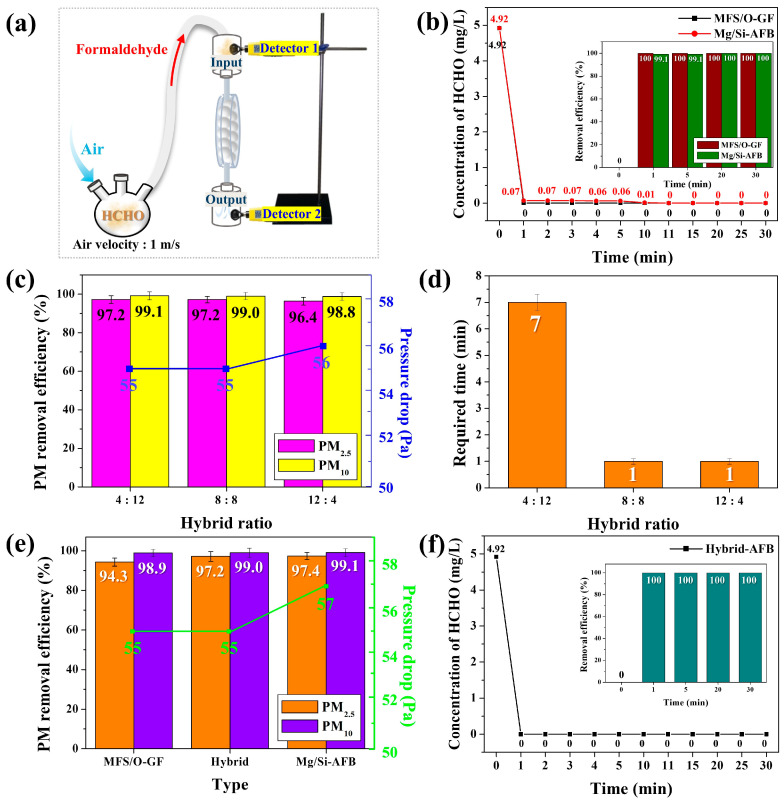
(**a**) Experimental setup of the disk-type air filtration system containing O-GF-AFBs or Mg/Si-AFBs for HCHO removal. (**b**) Variations in the HCHO gas concentration passing through the O-GF-AFBs or Mg/Si-AFBs as a function of time. The inset shows the corresponding REs. (**c**) HCHO REs and pressure drops of the air filtration systems with different hybrid ratios. (**d**) Time required to reach 0 ppm for the air filtration systems with different hybrid ratios. (**e**) PM REs and pressure drops of the different air filtration systems [O-GF-AFBs, Mg/Si-AFBs, and hybrid AFB (8:8)]. (**f**) Variations in the HCHO gas concentration passing through the hybrid AFB (8:8) as a function of time. The inset shows the corresponding REs.

To comparatively investigate the HCHO removal performance of the Mg/Si-AFB and O-GF-AFB, a disk-type air filtration system packed with 16 of each type of AFB was connected to the HCHO source chamber (left, ≤5 ppm) and two detectors (1, upper; and 2, lower) (Figure 5a). To perform the blank stage test, the variation in the HCHO gas concentration emitted from the chamber without AFBs was monitored. In the absence of the AFBs, the HCHO gas concentration was recorded as 0.11 ppm at 1 min and decreased to 0.01 ppm at 15 min (Figure S5). This decay suggests that HCHO was physically adsorbed onto the interior surface of the acrylate chamber when no filter was present.

The concentration of the HCHO gas emitted from the disk-type chamber—and thus, the residual HCHO concentration—was monitored by the lower detector (detector 2) as a function of time. As soon as the reaction began, the concentration of HCHO gas decreased sharply and reached 0 ppm within 1 min when using the O-GF-AFB (black line, Figure 5b). After reaching 0 ppm at 1 min, the concentration remained constant at this level for 30 min, indicating that the HCHO gas had been completely and irreversibly removed. The RE of HCHO for the O-GF-AFB was therefore 100% at 1 min (inset of Figure 5b). The concentration of HCHO gas also decreased sharply, reaching 0 ppm after 11 min with the Mg/Si-AFB (red line, Figure 5b). Once the concentration reached 0 ppm at 11 min, the HCHO gas was considered to have been completely and irreversibly removed. The RE for HCHO was 99.9% at 1 min and 100% at 11 min for the Mg/Si-AFB (inset of Figure 5b). Both types of air filters reduced the HCHO concentration to below the recommended indoor exposure limit (WHO standard, 0.08 ppm/m^3^) within 1 min. However, the Mg/Si-AFB required 11 min for complete removal, whereas the O-GF-AFB achieved complete removal within 1 min (Figure 5b). The adsorption behavior of HCHO gas on the O-GF-AFB was elucidated by FT-IR spectroscopy. Upon HCHO exposure, distinct vibrational bands emerged or intensified at 1457 cm^−1^ (–CH stretching), 1328 cm^−1^ (C–O stretching), 1075 cm^−1^ (–C–O–C– stretching), and 809 cm^−1^ (–CH stretching), corresponding to the characteristic absorptions of para-formaldehyde (Figure S6, red line) [29]. These observations indicate that the adsorbed HCHO molecules partially polymerized to para-HCHO on the surface of the O-GF-AFB, which is consistent with the known instability of gaseous formaldehyde [29].

Once polymerized, the para-HCHO species were strongly retained on the surface and could not be desorbed even under vacuum at room temperature, likely due to their larger molecular size and reduced volatility. In addition, the abundant surface hydroxyl groups of the O-GF-AFB enhanced the adsorption affinity toward polar VOCs such as HCHO, promoting the capture and stabilization of the adsorbed species [29]. The Mg/Si-AFB showed better performance in PM removal, while the O-GF-AFB was more effective in HCHO removal. Thus, hybrid AFBs composed of Mg/Si-AFBs and O-GF-AFBs were tested at different hybrid ratios to evaluate their PM and HCHO removal performance. The air filtration systems with different hybrid ratios (O-GF:Mg/Si = 4:12, 8:8, and 12:4) are referred to as hybrid AFB (4:12), hybrid AFB (8:8), and hybrid AFB (12:4), respectively. The hybrid AFBs with ratios of 4:12 and 8:8 exhibited nearly identical PM REs and pressure drops (Figure 5c). However, when the proportion of O-GF-AFBs increased [Hybrid AFB (12:4)], the REs decreased while the pressure drops increased (Figure 5c). These results indicate that a lower proportion of Mg/Si-AFBs leads to a deterioration in PM removal performance, and that a minimum Mg/Si-AFB content of 50% is required to achieve high PM RE. In the HCHO removal test, both hybrid AFB (8:8) and hybrid AFB (12:4) exhibited identical performance, completely removing HCHO within 1 min (Figure 5d). In contrast, hybrid AFB (4:12) required 7 min for complete HCHO removal (Figure 5d). These results indicate that a minimum of 50% O-GF-AFB content is essential for the rapid elimination of HCHO. Therefore, hybrid AFB (8:8), which represents an equal mixture of O-GF-AFB and Mg/Si-AFB, was identified as the optimal configuration for the simultaneous and efficient removal of both PM and HCHO. To accurately evaluate the performance of the optimized hybrid AFB (8:8), the REs of PM and HCHO were compared with those of the single-component AFBs, O-GF-AFB and Mg/Si-AFB. Compared with Mg/Si-AFB, which is specialized for PM removal, the hybrid AFB (8:8) exhibited superior overall performance, achieving comparable PM RE while maintaining a lower pressure drop (Figure 5e). Moreover, the hybrid AFB (8:8) demonstrated the same HCHO RE as the O-GF-AFB, which is specifically designed for HCHO removal (Figure 5f). These results demonstrate that combining O-GF-AFB and Mg/Si-AFB in a hybrid configuration effectively enhances the removal performance of both PM and HCHO by integrating their respective advantages and compensating for their individual limitations (Table 1). A comprehensive comparison of different air filters is summarized in Table 2 [30,31,32,33]. The data clearly indicate that the hybrid AFB (8:8) exhibits superior removal performance, especially when considering the required HCHO removal time.

**Figure 6:**
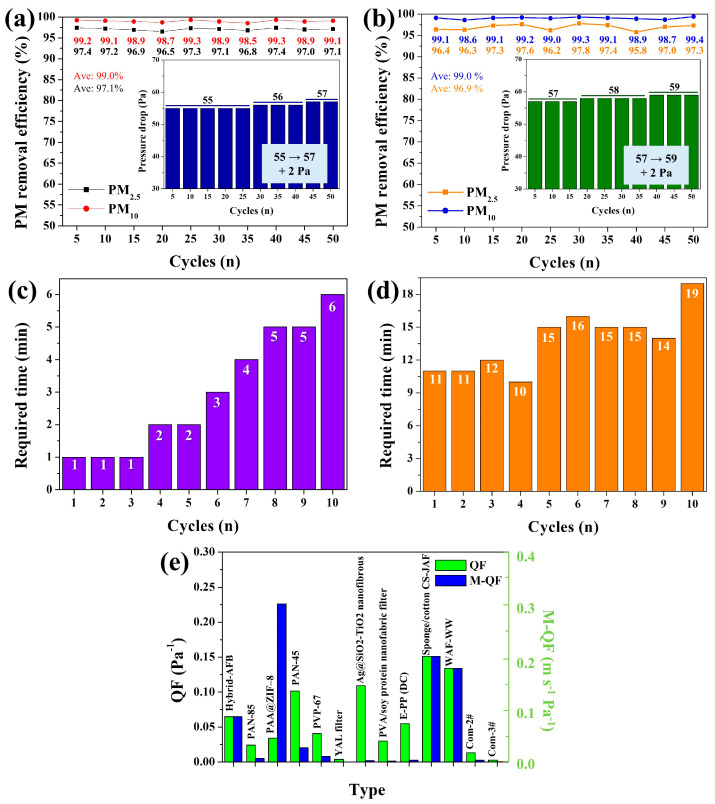
Long-term operation of the (**a**) hybrid AFB (8:8) and (**b**) Mg/Si-AFB systems for up to 50 cycles of PM removal. The insets show the corresponding pressure drops over 50 cycles. Long-term stability of the (**c**) hybrid AFB (8:8) and (**d**) Mg/Si-AFB systems for up to 10 cycles of HCHO removal. The time required to reach 0 ppm in each cycle by each system. (**e**) Comparison of QF and m-QF for different air filters.

The long-term performance of the hybrid AFB (8:8) was further evaluated to assess its practical applicability. For clarity, data points were recorded every 5 cycles during the 50-cycle test. The hybrid AFB (8:8) exhibited outstanding recyclability over 50 cycles, achieving the highest average PM_2.5_ removal efficiency (RE) of 97.1% (black line, Figure 6a). Notably, its performance stability was remarkable—the PM_2.5_ REs of the hybrid AFB (8:8) remained consistently high (97.4–96.5%) throughout 50 cycles. The variation between the maximum and minimum RE values (ΔRE = 0.9%) was negligible compared to that of the Mg/Si-AFB (ΔRE = 2.1%) (Figure 6a,b), confirming the superior durability and stability of the hybrid AFB (8:8). The pressure drop is one of the most critical factors influencing the practical applicability of air filters. Therefore, the pressure drop of each filter was also measured after every cycle during the long-term test. For the hybrid AFB (8:8), the pressure drops remained between 55 and 57 Pa throughout 50 cycles at an air velocity of 1 m/s (inset of Figure 6a). Specifically, the pressure drops were 55 Pa and 57 Pa after the 1st and 50th cycles, respectively, indicating only a 2 Pa increase over the entire testing period. The Mg/Si-AFB exhibited a pressure drop range of 57–59 Pa (ΔP = 2 Pa), comparable to that of the hybrid AFB (8:8) (55–57 Pa, ΔP = 2 Pa) (inset of Figure 6b). However, the overall pressure drops for the Mg/Si-AFB system were higher, which can be attributed to the greater proportion of Mg/Si-AFB present. These results demonstrate that the hybrid AFB (8:8) exhibits outstanding recyclability, maintaining an average PM_2.5_ RE of 97.1% over 50 cycles, with superior performance stability (ΔRE_min–max_ = 0.9%) and lower pressure drops (55–57 Pa) compared to the Mg/Si-AFB. Long-term performance and regeneration tests of the hybrid AFB (8:8) system were conducted at high PM concentrations (641,000 g/m^3^) for up to 35 cycles. The hybrid system exhibited PM_2.5_ REs ranging from 99.4% to 99.8% at this high concentration up to the 25th cycle (Figure S7). These exhibited PM_2.5_ REs subsequently decreased slightly to 98.7% at the 30th cycle. After the 30th cycle, the hybrid AFB (8:8) was washed with DI water to remove accumulated PM particles and subsequently re-tested for PM removal. The hybrid system exhibited PM_2.5_ RE of 99.5% at the 35th cycle, a value analogous to those obtained during the initial 25 cycles. These results clearly demonstrate that the hybrid AFB (8:8) system maintains consistent performance even after regeneration and reuse.

Long-term performance tests were conducted at low HCHO concentrations below 5 ppm. The hybrid AFB (8:8) maintained 100% HCHO RE up to the 10th cycle (Figure 6c). For all initial HCHO concentrations, the levels dropped sharply after filtration with the hybrid AFB (8:8), reaching 0 ppm within 1 min or, at most, 6 min, demonstrating that the hybrid AFB (8:8) retained complete removal performance through 10 consecutive cycles (Figure 6c). In contrast, filtration with Mg/Si-AFB also reduced the initial HCHO concentrations to 0 ppm, but within a longer time range of 11–19 min, indicating a slower removal performance compared with the hybrid AFB (8:8) (Figure 6d). Overall, the hybrid AFB (8:8) exhibited outstanding recyclability (HCHO RE_at 1–6 min_ = 100%) over 10 cycles, along with rapid and complete removal of low-concentration HCHO gas (<5 ppm), confirming its excellent performance for HCHO gas purification. To evaluate the overall performance of the hybrid AFB (8:8), the quality factor (QF), QF = −ln(1 − E)/ΔP) and the modified quality factor (m-QF), m-QF = −ln(1 − E)·V/ΔP), which accounts for the airflow rate, were employed, where E is the PM RE, ΔP is the pressure drop, and V is the air velocity. After long-term tests of PM and HCHO removal, the surface morphology of the hybrid AFB was monitored. Following 50 reuse cycles, the pores of the hybrid AFB were significantly blocked with PMs, and the surface of the AFB skeleton became coated with PM, leading to the formation of a rough morphology (Figure S8). Furthermore, the pore size of the hybrid AFB remarkably decreased after HCHO filtration up to the 10th cycle, and the surface of the AFB skeleton was observed to be coated with para-HCHO (Figure S8). These results demonstrate the effective filtration capability of the hybrid AFB for both PM and HCHO. A comprehensive comparison of different air filters is summarized in Figure 6e and Figure S9. The data clearly indicate that the hybrid AFB (8:8) exhibits superior air filtration performance considering ΔP, V, QF, and m-QF. Furthermore, its QF and m-QF values are comparable to the best results reported in the literature, demonstrating the hybrid AFB (8:8)’s effectiveness and rapid capability in purifying PM particles. Because the system employs a flexible sponge ball–filling structure, it can be designed and manufactured as a customized air filtration system specialized for PM removal, VOC removal, or combined PM/VOC removal, depending on the characteristics of the filled AFBs. Furthermore, since it is a filling-type system, the packing density within the air filter chamber can be readily adjusted, enabling easy control of both RE and pressure drop by varying the number of AFBs. Unlike typical microfibrous/nanofiber membranes-whose filtration performance is mainly determined by fixed pore size, fiber diameter, and membrane thickness-our AFB-based platform operates through a 3D-packed geometry that enables dynamic flow redistribution and tunable packing density. This unique design leads to several key performance differences. Compared with conventional microfibrous or nanofiber filters, the packed-type-AFB system demonstrates distinct advantages, including (i) stable PM_2.5_ RE even at high air velocities (up to 7 m/s), (ii) lower pressure-drop increases due to its 3D porous packing architecture, and (iii) superior recyclability over 50 cycles. Furthermore, micro/nanofiber filters have fixed structural configurations once fabricated. Our system allows real-time control of the number of AFBs, enabling adjustment of both RE and pressure drop without altering the filter material itself. This reconfigurability is not achievable with conventional membranes. The hybrid AFB system also achieved superior dual-functionality through the modular AFB design. The system’s performance is tunable via modular AFB design and adjustable packing density, allowing for customization to target specific pollutants, such as PM, HCHO, or a combination of both. These features highlight the uniqueness of the AFB-based filtration platform relative to conventional fiber-based filtration materials. Because the filter performance can be tuned by adjusting the number and ratio of AFBs (Mg/Si-AFB vs. O-GF-AFB), it is appropriate for customizable cartridge-type filters in compact ventilation units, portable purifiers, and smart HVAC systems. The AFB system maintains high PM RE even at high air velocities (up to 7 m/s), making it applicable to factories, workshops, and point-of-source ventilation where strong airflow is unavoidable.

## 4. Conclusions

A novel packed-type flexible AFB system was successfully fabricated for the simultaneous removal of PM and HCHO. The Mg/Si-AFB, synthesized through sequential modification of MFS, GF oxidation, and Mg–Si embedding, exhibited high PM RE (97.4%) and stable performance even at high air velocities due to its unique internal structure and flow distribution characteristics. The O-GF-AFB showed excellent HCHO removal capability, achieving complete elimination within 1 min. By integrating Mg/Si-AFBs and O-GF-AFBs at an optimal ratio (8:8), the hybrid AFB system achieved superior dual-functionality, maintaining high REs for both PM (97.1%) and HCHO (100%) while preserving low pressure drops and outstanding recyclability over 50 cycles. The hybrid AFB (8:8) demonstrated high QF and m-QF values, confirming its superior balance between filtration efficiency and aerodynamic resistance. Owing to its flexible sponge ball–filling design, this air filtration platform enables easy tuning of packing density, removal performance, and pressure drop. Furthermore, it can be customized to target specific pollutants, such as PM, HCHO, or both, offering a scalable and sustainable solution for next-generation air purification systems. The practical potential of this approach is significant, as the packed configuration allows flexible assembly for different environments, easy maintenance, and scalable adaptation to residential and industrial applications. However, this work is limited by the lack of long-term cycling and fouling studies, as well as the absence of large-scale prototype testing under real-world pollutant mixtures. Future studies will focus on durability evaluation, optimization of bead functionalization for multi-pollutant removal, and the development of automated control strategies for dynamic adjustment of packing density during operation.

## Figures and Tables

**Table 1:** Summary of PM and HCHO removal performance of MFS/GF, MFS/O-GF, and MFS/O-GF/Mg–Si, and hybrid AFB (8:8).

Filter Type	PM_2.5_ RE (%)	PM_10_ RE (%)	HCHO RE (%)	Time to Reach 0 ppm (HCHO)
MFS/GF	93.8	98.6	Not applicable	Not applicable
MFS/O-GF	94.3	98.9	100	1 min
MFS/O-GF/Mg–Si (Mg/Si-AFB)	97.4	99.1	100	11 min
Hybrid AFB (8:8)	97.2	99.0	100	1 min

**Table 2:** A summary of representative literature values.

Material Type	HCHO Removal Time	Operating Conditions	Reference Examples
laminated plate	8 min	0.8–1.2 m/s, low ΔP (5 Pa)	[30]
1%Ag-TiO_2_-silk	150 min	low ΔP (34.3 Pa)	[31]
Eco Excel with PAH (Running water)	40 min	1.58 m/s	[32]
photocatalytic coating material (TiO_2_)	12 min	1 m/s	[33]
O-GF-AFB	1 min (100% removal)	1 m/s, low ΔP (55–57 Pa)	This work
Hybrid AFB (8:8)	1–6 min (complete removal)	1 m/s, low ΔP (55–57 Pa)	This work

## Data Availability

The original contributions presented in this study are included in the article/Supplementary Material. Further inquiries can be directed to the corresponding author.

## Supplementary Materials

The following supporting information can be downloaded at: https://www.mdpi.com/article/10.3390/polym17243312/s1, Figure S1: BET analysis of nitrogen adsorption isotherm curves; Figure S2: XRD pattern of MFS/O-GF/Mg-Si (Mg/Si-AFB); Figure S3: Packing density of the Mg/Si-AFB as a function of the number of AFBs; Figure S4: PM_2.5_ REs of the air filtration system loaded with 12 and 16 Mg/Si-AFBs at air velocities of 1 m/s and 7 m/s; Figure S5: HCHO removal performances of the air filtration system without AFBs for the blank stage test; Figure S6: FT-IR spectroscopy data of the O-GF-AFB before and after HCHO filtration.; Figure S7: Long-term performance and regeneration tests of the hybrid AFB (8:8) system; Figure S8: SEM images of the hybrid AFBs before and after long-term tests of PM and HCHO removal; Figure S9: References for QF and m-QF comparison of different air filters [13,15,23,34,35,36,37,38,39].
