# Supplementary material for: A Packed-Type Reconfigurable Air Filtration System for Removal of Particulate Matter and HCHO
Source: Polymers (Basel). 2025 Dec 15;17(24):3312. doi: 10.3390/polym17243312 (PMC12736461; doi:10.3390/polym17243312)
Supplement: Supplementary file 1 [file polymers-17-03312-s001.zip › polymers-3989525-supplementary.pdf]

Supporting Information

A Packed-Type Reconfigurable Air Filtration System for Removal of Particulate Matters and HCHO

*Eun Jin Kim, Seung Hee Han, Dong Geon Lee, and Won San Choi**

Department of Chemical and Biological Engineering, Hanbat National University, 125 Dongseodaero, Yuseong-gu, Daejeon 305-719, Republic of Korea, E-mail: choiws@hanbat.ac.kr

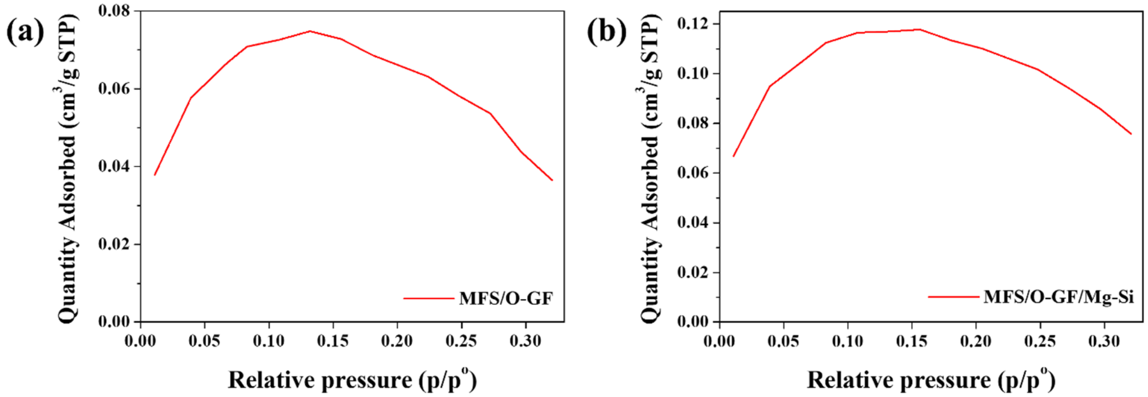

	BET surface area (m²/g)	R^2
**MFS/O-GF**	0.3145	0.99888
**MFS/O-GF/Mg-Si**	0.4894	0.99934

Figure S1. BET analysis of nitrogen adsorption isotherm curves: (a) MFS/O-GF and (b) MFS/O-GF/Mg-Si.

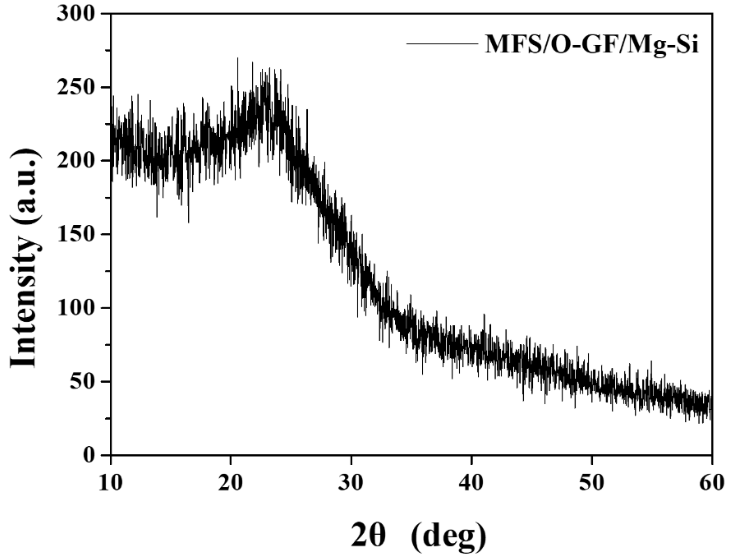

Figure S2. XRD pattern of MFS/O-GF/Mg-Si (Mg/Si-AFB).

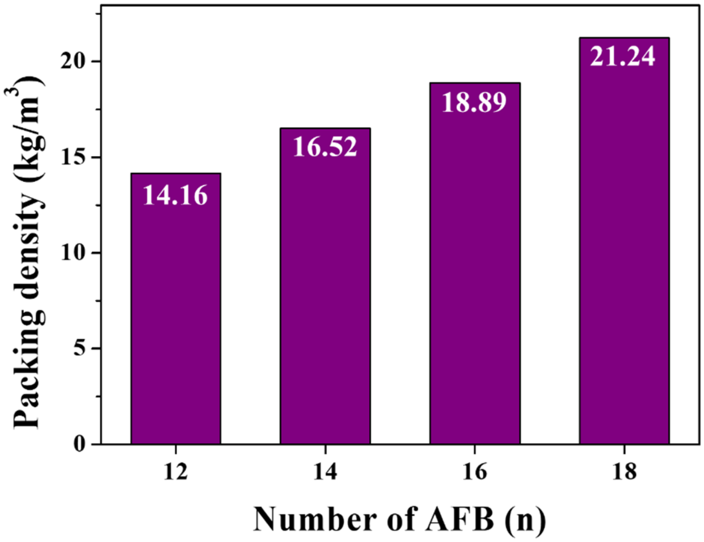

Figure S3. Packing density of the Mg/Si-AFB as a function of the number of AFBs.

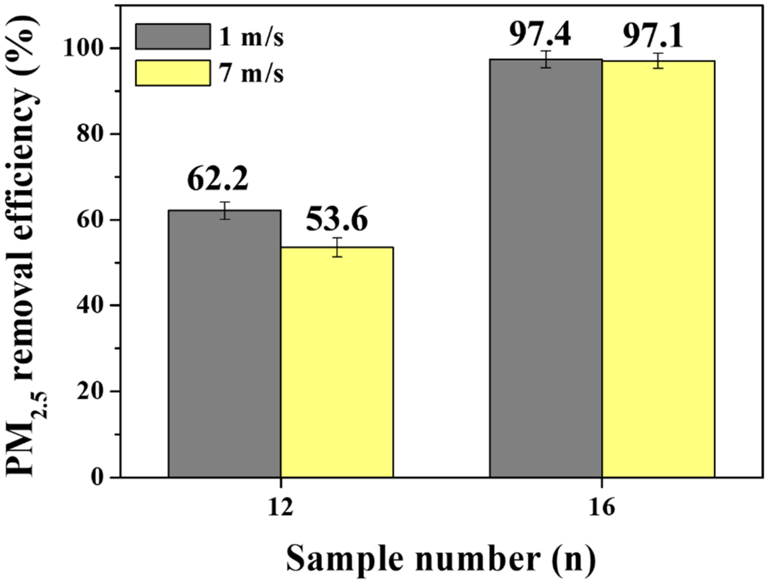

Figure S4. $PM_{2.5}$ REs of the air filtration system loaded with 12 and 16 Mg/Si-AFBs at air velocities of 1 m/s and 7 m/s.

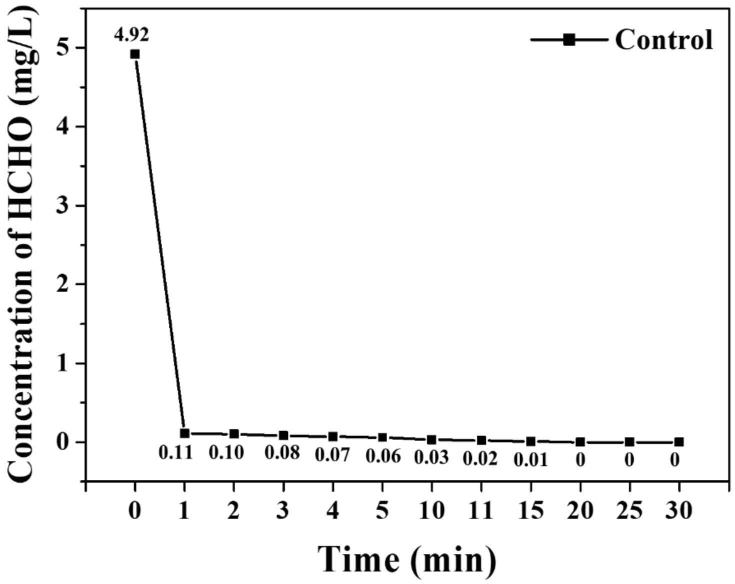

Figure S5. HCHO removal performances of the air filtration system without AFBs for the blank stage test.

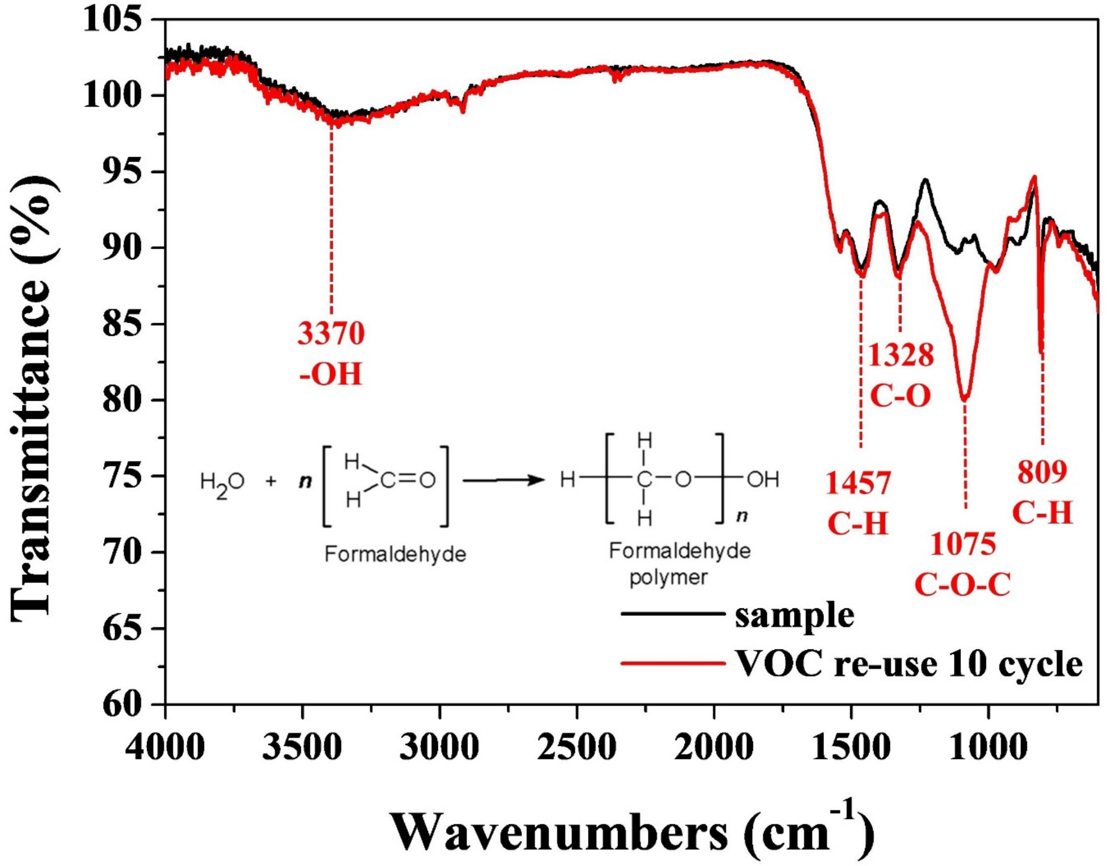

Figure S6. FT-IR spectroscopy data of the O-GF-AFB before and after HCHO filtration.

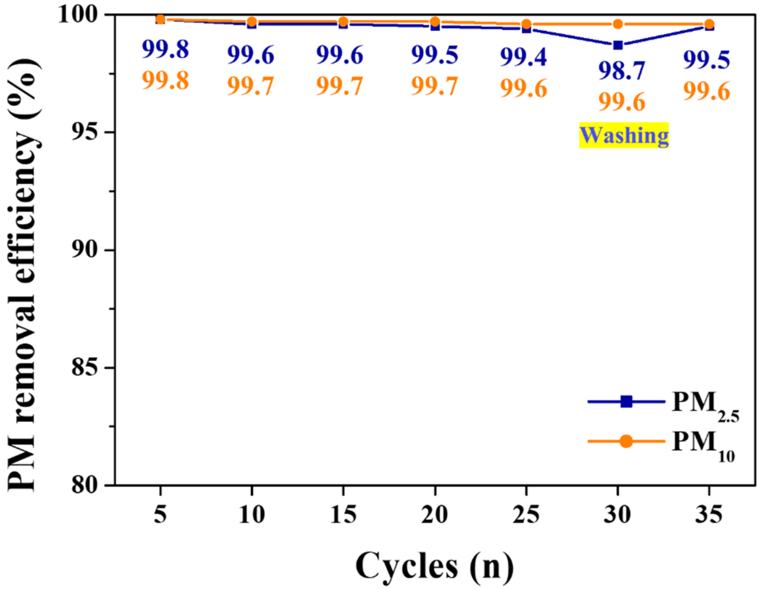

Figure S7. Long-term performance and regeneration tests of the hybrid AFB (8:8) system.

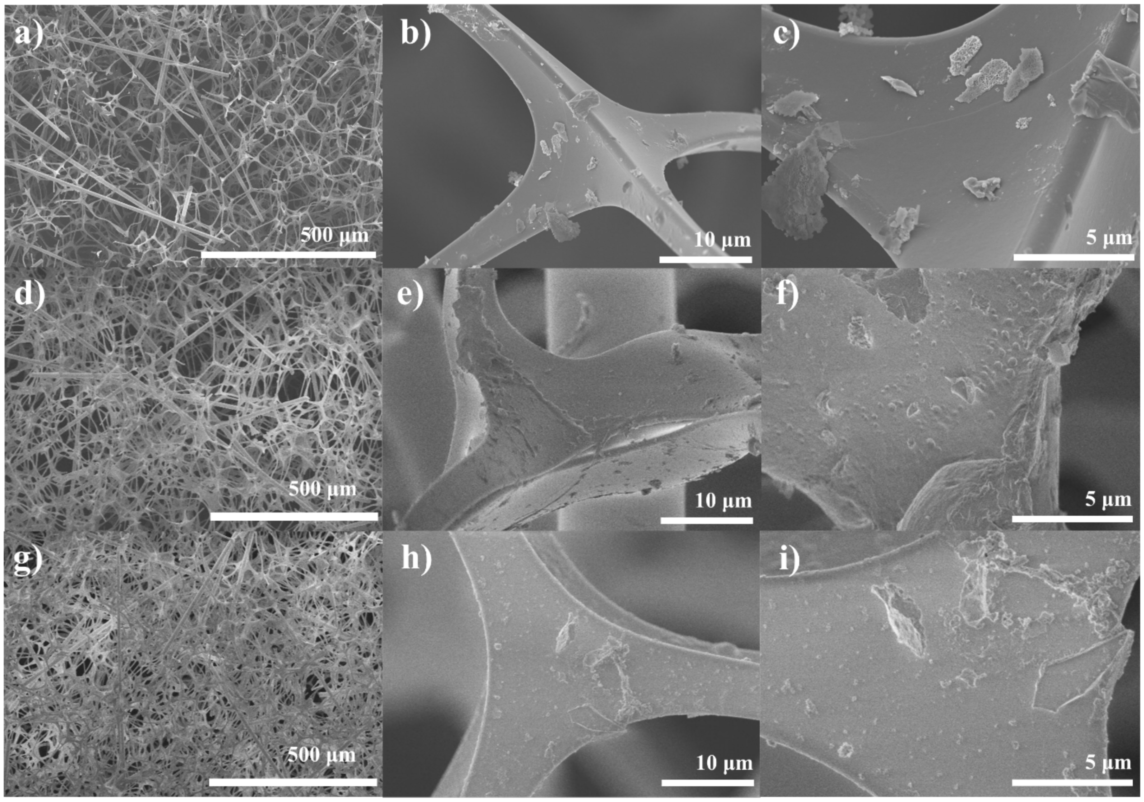

Figure S8. SEM images of the hybrid AFBs: (a-c) before and (d-i) after long-term tests of PM and HCHO removal. Specifically, (d-f) shows results after PM removal tests, and (g-i) show results after HCHO removal tests.

Sample	ΔP (Pa)	Air Velocity (m s^{-1})	E (%)	QF (Pa^{-1})	M-QF (m s^{-1} Pa^{-1})	Ref.
Hybrid-AFB	55	1	97.2	0.065	0.065	Our work
PAN-85	133	0.21	96.12	0.024	0.00513	(15)
PAA@ZIF–8	146.3	6	99.6	0.034	0.226	(34)
PAN-45	80	0.2	99.97	0.1014	0.0203	(35)
PVP-67	71	0.2	94.43	0.0407	0.00813	(35)
YAL filter	167	0.053	45.8	0.0037	0.00019	(36)
Ag@SiO_2-TiO_2 nanofibrous	59	0.02	99.84	0.109	0.0022	(37)
PVA/soy protein nanofabric filter	215	0.05	99.8	0.03	0.00144	(38)
E-PP (DC)	39.72	0.05	88.78	0.055	0.00275	(39)
Sponge/cotton CS-JAF	61	1	99.99	0.151	0.151	(13)
WAF-WW	26	1	96.9	0.134	0.134	(23)
Com-2#	499	0.2	99.87	0.0133	0.00266	(35)
Com-3#	243	0.2	49.66	0.0028	0.00056	(35)

Figure S9. Table and references for QF and m-QF comparison of different air filters.

13. Park, S.; Koo, H.Y.; Yu, C.; Choi W.S. A novel approach to designing air filters: Ubiquitous material-based Janus air filter modules with hydrophilic and hydrophobic parts.

Chem. Eng. J. **2021**, *410*, 128302.

15. Liu, C.; Hsu, P.C.; Lee, H.W.; Ye, M.; Zheng, G.; Liu, N.; Li, W.; Cui, Y. Transparent air filter for high-efficiency $PM_{2.5}$ capture. *Nat. Commun.* **2015**, *6*, 6205.

23. Jung, Y.J.; Son, S.H.; Choi, W.S. Density-gradient water-based air filters: Gas/solid-permeable and liquid-impermeable design. *Separ. Purif. Technol.* **2024**, *343*, 127150.

34. Guo, J.; Hanif, A.; Shang, J.; Deka, B.J.; Zhi, N.; An, A.K. PAA@ZIF-8 incorporated nanofibrous membrane for high-efficiency $PM_{2.5}$ capture. *Chem. Eng. J.* **2021**, *405*, 126584.

35. Zhang, R.; Liu, C.; Hsu, P.C.; Zhang, C.; Liu, N.; Zhang, J.; Lee, H.R.; Lu, Y.; Qiu, Y.; Chu, S.; Cui, Y. Nanofiber Air Filters with High-Temperature Stability for Efficient $PM_{2.5}$ Removal from the Pollution Sources. *Nano Lett.* **2016**, *16*, 3642–3649.

36. Suriaman, I.; Hendrarsakti, J.; Mardiyati, Y.; Pasek, A. D. Synthesis and characterization of air filter media made from cellulosic ramie fiber (Boehmeria nivea). *Carbohydrate Polymer Technol. Appl.* **2022**, *3*, 100216.

37. Wang, B.; Zhang, Y.; Wang, X.; Zhang, X.; Liu, Y.; Zhang, Y.; Zhang, H.; Zhang, Y. Flexible Multifunctional Porous Nanofibrous Membranes for Efficient Air Filtration. *ACS Appl. Mater. Interfaces.* **2019**, *11*, 43409-43415.

38. Souzandeh, H.; Johnson, K. S.; Wang, Y.; Bhamidipaty, K.; Zhong, W.H. Soy-Protein-Based Nanofabrics for Highly Efficient and Multifunctional Air Filtration. *ACS Appl. Mater. Interfaces.* **2016**, *8*, 20023–20031.

39. Mercier, C.; Kirsch, R.; Antonyuk, S. Analytical model for the initial efficiency of compressed nonwoven electret media for air filtration. *Chem. Eng. Res. Des.* **2025**, *216*,

549-563.
